# Leaf UV-B Irradiation and Mycorrhizal Symbionts Affect Lettuce VOC Emissions and Defence Mechanisms, but Not Aphid Feeding Preferences

**DOI:** 10.3390/insects14010020

**Published:** 2022-12-24

**Authors:** Valeria Zeni, Arianna Grassi, Marco Santin, Renato Ricciardi, Ylenia Pieracci, Guido Flamini, Filippo Di Giovanni, Margherita Marmugi, Monica Agnolucci, Luciano Avio, Alessandra Turrini, Manuela Giovannetti, Monica Ruffini Castiglione, Annamaria Ranieri, Angelo Canale, Andrea Lucchi, Evgenios Agathokleous, Giovanni Benelli

**Affiliations:** 1 Department of Agriculture, Food and Environment, University of Pisa, Via del Borghetto 80, 56124 Pisa, Italy; 2 Department of Pharmacy, University of Pisa, Via Bonanno 6, 56126 Pisa, Italy; 3 Interdepartmental Research Center Nutrafood—Nutraceuticals and Food for Health, University of Pisa, 56124 Pisa, Italy; 4 Department of Life Science, University of Siena, Via Aldo Moro 2, 53100 Siena, Italy; 5 Department of Biology, University of Pisa, Via L. Ghini 13, 56126 Pisa, Italy; 6 Department of Ecology, School of Applied Meteorology, Nanjing University of Information Science & Technology (NUIST), Nanjing 210044, China

**Keywords:** AMF, chemical ecology, lipid peroxidation, *Myzus persicae*, stomatal density, ROS, volatile organic compounds

## Abstract

**Simple Summary:**

Arbuscular mycorrhizal fungi (AMF), as well as ultraviolet-B radiation (UV-B), could act as key factors affecting plant–insect interactions. The stress related to one of these two factors could be balanced by the positive effects of the other. In the present work, the feeding preferences of *Myzus persicae* aphids for lettuce plants exposed to two abiotic factors, i.e., AMF and UV-B, singly or in combination, was evaluated. Results showed that lettuce plants treated with UV-B increased in callose and oxidative stress indicators, signalling an alteration in the volatile organic compounds (VOCs) emitted. On the other hand, the negative effects of UV-B were partially mitigated in lettuces inoculated with AMF. Differently treated lettuces did not affect the feeding preferences of the *M. persicae* aphid population. Overall, this study shed light on how UV-B and AMF impact lettuce VOCs emission and defence mechanisms, but not the feeding behaviour of a polyphagous aphid species.

**Abstract:**

Arbuscular mycorrhizal fungi (AMF) and ultraviolet-B radiation (UV-B) play important roles in plant–insect interactions by altering plant physiology and histology. We hypothesized that UV-B-induced oxidative stress was mitigated by AMF symbiosis. In this study, we conducted a multifactorial experiment to explore lettuce plant response to AMF inoculation and UV-B exposure (0.4 W m^−2^; 16 h d^−1^; 2 weeks), either together or individually, as well as the interaction with the polyphagous insect pest *Myzus persicae* (Sulzer). Lettuce plants subjected to UV-B radiation showed an increase in callose and oxidative stress indicators, as well as a decrease in stomatal density. Mycorrhizal colonization cancelled out the effect of UV-B on stomatal density, while the symbiosis was not affected by UV-B treatment. The plant volatile emission was significantly altered by UV-B treatment. Specifically, the non-terpene 1-undecene abundance (+M/+UVB: 48.0 ± 7.78%; −M/+UVB: 56.6 ± 14.90%) was increased, whereas the content of the non-terpene aldehydes decanal (+M/+UVB: 8.50 ± 3.90%; −M/+UVB: 8.0 ± 4.87%) and undecanal (+M/+UVB: 2.1 ± 0.65%; −M/+UVB: 1.20 ± 1.18%) and the sesquiterpene hydrocarbons (+M/+UVB: 18.0 ± 9.62 %; −M/+UVB: 19.2 ± 5.90%) was decreased. Mycorrhization, on the other hand, had no significant effect on the plant volatilome, regardless of UV-B treatment. Aphid population was unaffected by any of the treatments, implying a neutral plant response. Overall, this study provides new insights about the interactions among plants, UV-B, and AMF, outlining their limited impact on a polyphagous insect pest.

## 1. Introduction

Ultraviolet-B (UV-B) radiation (280–315 nm)—a small fraction of solar radiation reaching the Earth’s surface—represents an important light signal responsible for the development of plant-specific photomorphogenic responses [1,2]. Increased levels of UV-B radiation can affect the morphology and the physiological processes of plants, as well as alter the performance and preference of herbivorous arthropods. At low, ecologically relevant doses, UV-B radiation enhances the plant defences by increasing the production of secondary metabolites without determining an overproduction of reactive oxygen species (ROS) and/or lethal impairments in the photosynthetic apparatus [3,4]. In light of these positive effects of low UV-B doses, several studies have investigated the priming effect of UV-B exposure towards several abiotic stresses occurring afterwards [5,6,7,8,9]. However, UV-B radiation may also indirectly affect arbuscular mycorrhizal fungi (AMF) through morphological, physiological, and biochemical modifications of the host plants [10,11,12]. AMF are widely spread in natural and agricultural ecosystems, and their symbiotic relationship with most land plants provides important ecological services, e.g., increasing plant nutrition and tolerance to both abiotic and biotic stresses, including herbivorous pests [13]. The mycorrhizal status of plants is also a key factor leading to changes in plant physiology, e.g., nutrient dynamics and secondary metabolism [14,15,16,17], thus affecting the quality of plants as suitable hosts for insect pests [18]. AMF and UV-B may be involved in the modification of the emission of volatile organic compounds (VOCs), which are responsible for the communication of plants with the surrounding environment [19,20]. For instance, treatment with elevated UV-B doses increased the yields of essential oils in two *Curcuma* species, *C. caesia* and *C. longa* [21]. Moreover, variations in the production of *Eclipta alba* essential oil were found to be dependent on UV-B exposure method [22]. As regards AMF, changes in VOCs production have been reported for leaves of some plant species, such as *Artemisia annua*, *A. umbelliformis*, *Bituminaria bituminosa*, *Ocimum basilicum*, and *Vitis vinifera,* colonized by different fungal symbionts [23,24,25,26,27,28].

VOCs can cross several trophic levels, acting as indirect plant defences by guiding natural enemies to locate their herbivore prey on plants, thereby minimizing herbivore damage [29,30]. Herbivores, on the other hand, identify appropriate hosts using volatile cues [31,32]. VOCs play a role in many ecological networks and can benefit the plants that can best exploit them [29]. Therefore, it is mandatory to investigate the potential impacts of UV-B and AMF treatments on VOCs emissions in relation to insect pest infestation. The current study seeks to shed light on the potential impacts of UV-B radiation and AMF colonization on lettuce (*Lactuca sativa* L.), a valuable horticultural crop, by assessing the individual and combined effects of AMF and UV-B on stomatal density (SD), VOCs emission, and oxidative stress of lettuce plants. We investigated how the treatments affected the trophic activity and fitness of green peach aphids, *Myzus persicae* (Sulzer) (Hemiptera: Aphididae), a widespread pest of horticultural crops. *Myzus persicae* infestations, as well as other aphid species, can cause direct plant damage through the transmission of various hazardous phytopathogens, including the lettuce mosaic virus [33,34,35]. To achieve high lettuce quality and productivity, eco-friendly aphid control strategies must be devised. We hypothesized the induction of mild oxidative stress through UV-B, therefore arguing a possible mitigation of this effect by AMF symbiosis. In this context, the current research aims to determine if UV-B and/or AMF-induced morphological, physiological, and biochemical alterations in lettuce might boost tolerance to the polyphagous aphid *M. persicae*. 

## 2. Materials and Methods

### 2.1. Insect Rearing

The colony of *M. persicae* was reared in laboratory conditions [27 ± 1 °C, 70 ± 5% R.H., and 16:8 (L:D) photoperiod] on pea plants (*Pisum sativum*, at the Entomology Lab of the Department of Agriculture, Food and Environment (DAFE), University of Pisa (Italy) ) in insect cages, following the protocol reported by Canovai et al. [36].

### 2.2. Plant Material and Treatments

Organic seeds of red leaf lettuce (*L. sativa* L.) cv. Red Salad Bowl, purchased from Landen company (Blumen Group, Milan, Italy), were germinated on moisturized filter paper in appropriate climatic chambers and kept in the dark at 24 °C for 72 h, followed by a 16:8 (L:D) h photoperiod. Five days after sowing, 120 sprouts with fully developed cotyledons were transferred onto a polystyrene plug tray (16 mL per cell, one sprout per cell) and inoculated with 50% (*v*/*v*) of crude inoculum (mycorrhizal roots and soil containing spores and extraradical mycelium) of the AMF species *Funneliformis mosseae* (T.H. Nicolson & Gerd.) C. Walker & A. Schüßler, isolate IMA1, obtained from *Trifolium alexandrinum* pot-cultures, kept in the Microbiology Laboratories’ collection (International Microbial Archives, IMA) of the Department of Agriculture, Food and Environment, University of Pisa. Other 120 sprouts, cultivated in sterilized calcined clay, were provided with steam-sterilized crude inoculum (mock inoculum) and 2 mL of a filtrate made by filtering mycorrhizal inoculum through a 50-µm pore diameter sieve and Whatman paper no. 1 (Whatman International Ltd., Maidstone, Kent, UK) to assure the same microbiota to all treatments. Plantlets were watered with distilled water (10 mL per pot), as needed, and fertigated with a modified half-strength Hoagland’s nutrient solution (pH 6, 1.15 mS/cm EC) twice a week in growth chambers. Blue/red (1:2 ratio) and green (10%) LEDs (C-LED, Imola, Italy) provided photosynthetic active radiation (PAR) with a photosynthetic flux density (PPFD) of 228 mol m^−2^s^−1^. After one week of cultivation in the growth chambers at 24 °C with a 16:8 (L:D) h photoperiod, mycorrhizal (+M) and non-mycorrhizal (−M) plantlets were exposed to UV-B LEDs (C-LED, Imola, Italy) for two weeks. The plantlets were exposed to 0.4 W m^−2^ for 16 h a day (23.04 kJ m^2^ of UV-B). The UV-B irradiation overlapped the photoperiods 16 h of light. Separate plastic trays were used for +M and −M plants to prevent mycorrhizal fungal cross-contamination. At the end of UV-B treatment, plants from all four groups (−M/−UVB, −M/+UVB, +M/−UVB, +M/+UVB) were randomly divided and sampled for microbiological, morphological, and biochemical analyses.

### 2.3. Analysis of AMF Root Colonization

The percentage of AMF colonization on the whole root systems of five replicate plants was determined for each treatment. Roots were washed in tap water, clarified in 10% KOH at 80 °C for 10 min, neutralized in 2% HCl, and dyed in lactic acid with 0.05% Trypan Blue. The proportion of colonized root length was calculated using the gridline intersect method on each root sample using a dissecting microscope (Wild, Leica, Milano, Italy) [37]. Colonized roots were selected, placed on microscope slides, and examined with a Reichert–Jung (Wien, Austria) Polyvar light microscope to discover intraradical fungal formations.

### 2.4. Stomatal Density

Epidermal leaf impressions were obtained from the tip and marginal edges of the abaxial side of young, developed leaves from five samples per treatment. The impressions were achieved using transparent nail polish, and, once dried, were mounted with double-sided tape on a microscope slide [38]. For each impression, at least ten random microscope fields were selected and analysed using Leitz Diaplan equipped with a Leica DCF420 ccd camera. Stomatal density (SD) has been reported as the number of stomata per mm^2^ [39].

### 2.5. Histochemical Detection of Hydrogen Peroxide, Lipid Peroxidation, and Callose Deposition

Leaf discs of 10-mm diameter were obtained by punching holes in five plants for each treatment. Histochemical detection of hydrogen peroxides was achieved by dipping the leaf discs in 3,3′-diaminobenzidine (DAB) (Sigma-Aldrich, St. Louis, MO, USA) that is oxidized with hydrogen peroxide occurring in plant cell/tissues to generate dark-brown precipitates. Samples were first vacuum-infiltrated for 20 min in the staining solution (1 mg/mL DAB, pH 3.8) [40]. Then, they were incubated in the same solution overnight and whitened in 96% ethanol for 60 min at 65 °C. Finally, they were analysed under a WILD Heerbrugg M420 stereomicroscope, equipped with a Canon PowerShotS45 camera, to estimate the presence of brown precipitates. In situ determination of lipid peroxidation was performed with Schiff’s reagent [41] (VWR Chemicals BDH, Lutterworth, UK), which, because it binds to free aldehyde groups, can be considered a qualitative indicator of lipid peroxidation. Leaf discs were incubated with the dye for 60 min at room temperature; then, samples were bleached in 96% ethanol for 60 min at 65 °C. The samples were then analysed under light stereomicroscope, as detailed above, to evaluate the developed purple colour. Staining methods for oxidative stress markers were combined with image processing to conduct downstream analysis of the digitalized leaf images. Staining intensity of DAB and Schiff’ reagent was estimated using ImageJ software as arbitrary units. Callose deposition evaluation was also performed on leaf discs, whitened overnight in ethanol 96% then washed in 0.07 M phosphate buffer pH 9. The staining procedure was achieved with aniline blue dye (Sigma-Aldrich), solubilized in the same buffer (0.05% *w*/*v*) for 2 h at room temperature [42]. Leaf discs mounted on glass slides were analysed using an epifluorescence microscope LEICA DMLB, equipped with a LEICA DFC 7000T camera. Callose-mediated fluorescence was visualised using a DAPI filter set (excitation filter 390 nm; dichroic mirror 420 nm; emission filter 460 nm) [43].

### 2.6. Plant Volatile Organic Compounds (VOCs) Analysis

The samples spontaneous VOC emission was measured using headspace solid-phase microextraction (HS-SPME), using a Supelco PDMS fibre (100 μm) (Supelco analytical, Bellefonte, PA, USA) preconditioned according to the manufacturer’s instructions. All the samples (approximately 1 g each) were placed in a 100-mL glass beaker and sealed with aluminium foil for at least 30 min. The headspaces (HSs) were, therefore, sampled at room temperature for 60 min before the fibre was retracted into the needle and immediately injected into the GC-MS apparatus. Four replicates were used in the analyses. Quantitative analyses of relative peak areas between the same compounds in different samples were carried out.

Gas chromatography-electron impact mass spectrometry (GC-EIMS) analyses were carried out on an Agilent 7890B gas chromatograph (Agilent Technologies Inc., Santa Clara, CA, USA) equipped with an Agilent HP-5MS capillary column (30 m × 0.25 mm; coating thickness 0.25 μm) and an Agilent 5977B single quadrupole mass detector under the following analytical conditions: (i) oven temperature ramp from 60 to 240 °C at 3 °C/min; (ii) injector temperature of 250 °C; (iii) transfer line temperature of 240 °C; (iv) and carrier gas helium at 1 mL/min. The constituents were identified by comparing their retention periods with those of authentic samples when available, as well as their linear retention times relative to the *n*-hydrocarbon series C_8_-C_27_. VOCs were also identified by matching their mass spectral characteristics and retention indices using commercial libraries (NIST 14 and ADAMS 2007) and laboratory-developed mass spectral libraries created from pure substances and constituents of known composition commercial essential oils, as well as MS literature data [44,45,46,47,48].

### 2.7. Feeding Preferences of Myzus persicae 

Herein, a four-choice test was performed to evaluate the feeding preferences of *M. persicae* toward treated/untreated lettuce plants. To not influence aphid preferences, a randomized experimental design was applied. For each treatment, a plant was taken and transplanted in a polystyrene (PS) tray (14.5 cm × 10.5 cm × 10 cm). Then, a pea apex, bearing a variable number of aphids (~20–30), was put in the middle of the container. The assays were performed with 30 replicates. To measure the effectiveness of treatments on aphid feeding preferences, the growing colony was kept at laboratory condition [27 ± 1 °C, 70 ± 5% R.H. and 16:8 (L:D) photoperiod]. The number of living adults and nymphs was counted after three days of feeding; then, the presence of aphids around or on a lettuce plant was considered as a choice. Aphids still located in close proximity of the pea apex after 72 h, were counted as no choice (NC).

### 2.8. Statistical Analysis

Using the JMP 16 Pro software program (SAS Institute, Cary, NC, USA, 1989–2021), the entire chemical composition of the lettuce HSs was subjected to principal component analysis (PCA) and hierarchical cluster analysis (HCA) (SAS Institute, Cary, NC, USA). The PCA was performed on a 26 × 4 (26 compounds × 4 samples = 104 data) covariance data matrix, selecting the two highest PCs produced by linear regression on mean-centred, unscaled data to reduce the dimensionality of the multivariate data matrix while retaining most of the variance [49,50].

The chosen PC1 and PC2 explained 97.4% of the overall variance by covering 89.4% and 8.0% of the variance, respectively. Ward’s approach for unscaled, non-standardized data was used, utilizing squared Euclidean distances as a measure of similarity. The chemical components of the lettuce HSs were also submitted to the Similarity Percentage Test (SIMPER) with the Bray–Curtis distance/similarity measure, to identify compounds that contributed at least 1.00% of the dissimilarity between + UVB/−UVB and +M/−M samples. The statistical significance of the difference in relative abundances of selected chemicals was assessed using the F- or *t*-test, depending on whether the compounds had equal or unequal variances. The SIMPER, F-, and *t*-tests were run using Past 4.03 Software (Natural History Museum, Oslo, Norway) [51]. The SIMPER compounds were also subjected to a Student *t*-test (*p* < 0.05) to determine whether there were significant differences between the +UVB/−UVB and +M/−M samples. Finally, the chemical classes of all the samples examined were subjected to analysis of variance (ANOVA). Tukey’s HSD test was used to separate averages, with a *p* < 0.05 threshold used to determine the significance of differences between means. The *t*-test and ANOVA were both performed with the JMP 16 Pro software program.

A chi-square test (*p* = 0.05) was used to determine whether the *M. persicae* selection was random. Data were neither normally distributed (Shapiro-Wilk test, *p* < 0.01) nor homoscedastic (Levene’s test, *p* < 0.01), and differences in aphid choice were investigated using a Kruskal–Wallis test, followed by Steel–Dwass post-hoc test (*p* = 0.05). JMP 16 Pro was used for statistical analysis.

Stomatal density and histochemical data were subjected to one-way ANOVA, followed by Tukey’s HSD test (*p* = 0.05 as the threshold). One-way ANOVA was used to analyse lettuce root mycorrhizal colonization data using the program IBM SPSS statistics version 23 (IBM Corporation, Armonk, NY, USA).

## 3. Results

### 3.1. Mycorrhizal Status of Experimental Plants

**Figure 1:**
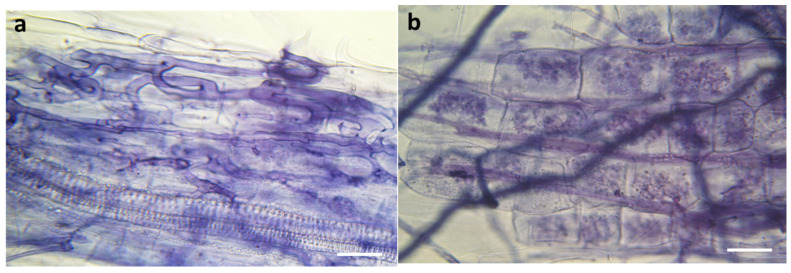
Light photomicrographs of fungal structures formed by *Funneliformis mosseae* IMA1 on the roots of *Lactuca sativa* var. Red Salad Bowl. (**a**) intraradical hyphae and coils showing the Arum-type colonization pattern; scale bars: 15 µm; (**b**) arbuscules produced within cortical root cells; scale bar: 10 µm.

At harvest, at the end of UV-B treatment (21 days post-inoculation), mycorrhizal roots of +UVB and −UVB lettuce plants did not show significantly different colonization (*p* = 0.08), reaching 38.0 ± 2.2% and 30.0 ± 3.4% (mean ± SE) of mycorrhizal root length in +UVB and −UVB plants, respectively. Control plants did not show any colonization, as expected, since they were mock inoculated. Both UV-B treated and untreated *F. mosseae* IMA1 inoculated plants showed an Arum-type colonization pattern, with AM hyphae spreading intercellularly among root cortical cells and many arbuscules forming terminally on intracellular hyphal branches (Figure 1).

### 3.2. Stomatal Density

**Figure 2:**
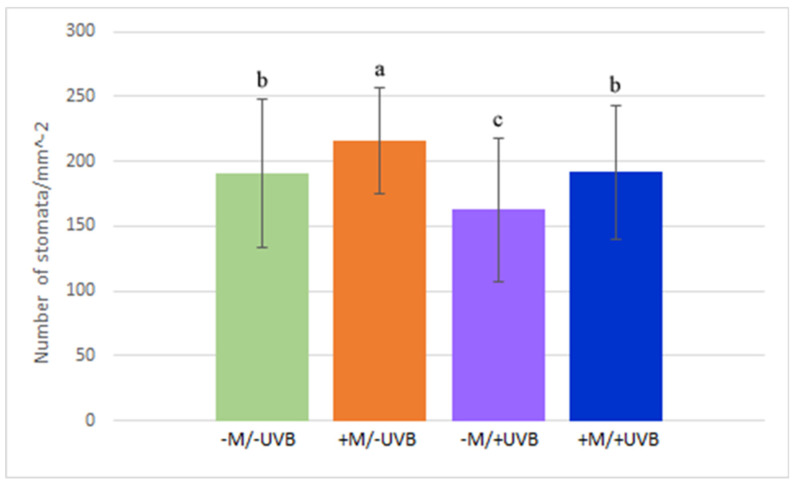
Histogram representing the mean values of stomatal density (±SD) in *Lactuca sativa* exposed or not to UV-B radiation (+UVB, −UVB), and colonized or not by arbuscular mycorrhizal fungi (+M, −M). Different letters indicate significant differences according to one-way ANOVA (*F*_3,245_ = 11.02, *p* < 0.001) followed by Tukey’s HSD test (*p* ≤ 0.05).

SD was significantly higher in mycorrhizal plants, whereas UV-B treatments led to a decrease in SD (Figure 2). Plants inoculated with AMF and treated with UV-B re-established a SD comparable to the control (Figure 2). 

### 3.3. Hydrogen Peroxide, Lipid Peroxidation, and Callose Deposition

**Figure 3:**
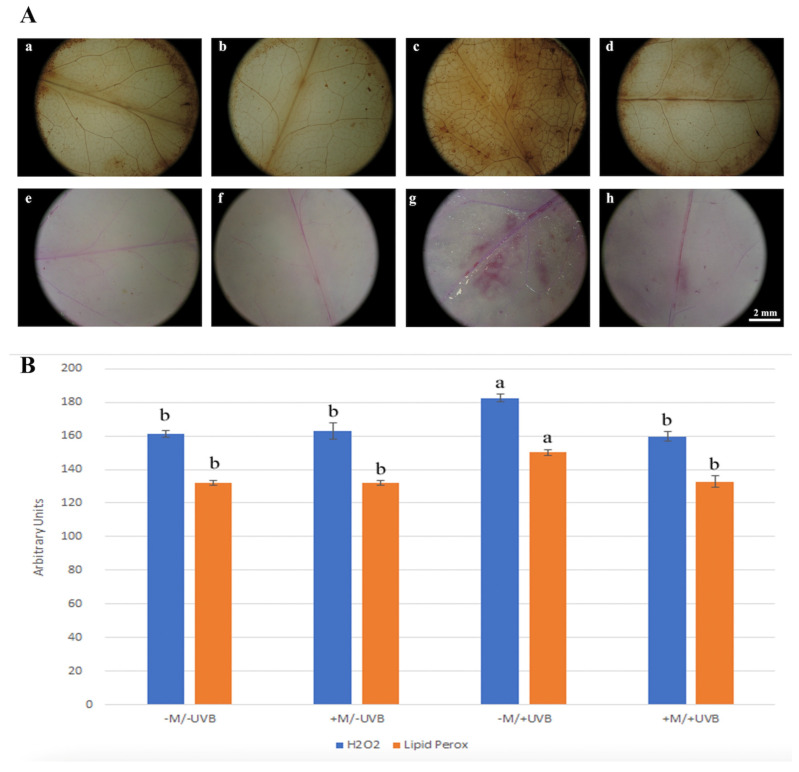
(**A**) Representative images of *Lactuca sativa* leaf discs exposed or not to UV-B radiation (+UVB, −UVB), and colonized or not by arbuscular mycorrhizal fungi (+M, −M), then processed for histochemical detection of oxidative stress markers. (**a**–**d**) DAB staining (H_2_O_2_ indicator); (**e**–**h**) Schiff ‘reagent staining (lipid peroxidation indicator). (**a**,**e**) −M/−UVB; (**b**,**f**) +M/−UVB; (**c**,**g**) −M/+UVB; (**d**,**h**) +M/+UVB. (**B**) Staining intensity of DAB and Schiff’ reagent estimated using ImageJ 1.53 software. Bars represent mean and error bars the standard deviation. Different letters indicate significant differences according to one-way ANOVA (DAB staining *F* = 55.03, *p* < 0.001; Schiff reagent staining *F* = 86.25, *p* < 0.001) and Tukey’s HSD test (*p* ≤ 0.05).

The histochemical approach was adopted to assess both oxidative stress marker production and distribution, as well as defence-related callose deposition, following the various treatments. The pattern of brown DAB-polymerization products, indicative of H_2_O_2_ formation, did not show specific differences in either intensity or distribution among the different treatments (Figure 3A(a,b,d),B), except for –M/+UVB treated plants. Microscopic analysis of –M/+UVB samples showed higher DAB responsiveness (Figure 3A(c),B), widespread throughout the leaf disc, where more dyed areas were distinguished. In situ determination of lipid peroxidation revealed a superimposable result compared to DAB staining (Figure 3A(e–h)). Schiff’s reagent produced a more intense response in –M/+UVB sample (Figure 3B), particularly marked in correspondence of the leaf main vein as well as in localized areas of leaf disc (Figure 3A(g)). Overall, UV-B treatment alone promoted an increase in oxidative stress markers, which were no longer detectable in +M-treated plants.

**Figure 4:**
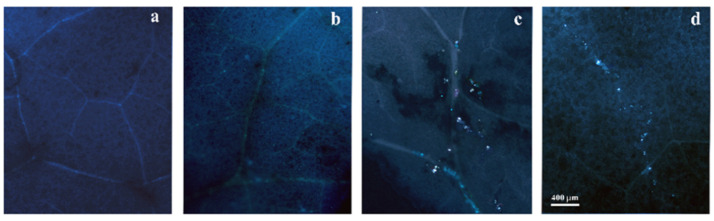
Representative images of *Lactuca sativa* leaves exposed or not to UV-B radiation (+UVB, −UVB), and colonized or not by arbuscular mycorrhizal fungi (+M, −M) following histochemical callose staining. (**a**) −M/−UVB; (**b**) +M/−UVB; (**c**) −M/+UVB; (**d**) +M/+UVB.

Callose deposits, as revealed by aniline blue dye, were barely detectable in −M/−UVB. However, the +UVB treatment, with and without mycorrhization, elicited callose deposition in some leaf areas, which appeared in the form of white precipitates (Figure 4c,d).

### 3.4. Plant Volatiles Profile

The complete chemical composition of the HSs of the analysed plants is reported in Table 1. Overall, 29 compounds were identified, covering 100% of the total compositions.

Non-terpene derivatives were the main chemical class of compounds detected in all the sample HSs, as they ranged between 62.4 and 76.7%. The samples exposed to UVB radiations showed higher amounts of this chemical class (70.1% in +M/+UVB, and 76.7% in −M/+UVB) than the unirradiated samples (69.9% in +M/–UVB and 62.4% in −M/−UVB). However, the differences in non-terpene percentages among all the samples were not statistically significant. Compounds 1-undecene and decanal were the most representative components belonging to this class, accounting for up to 56.6 and 27.4%, respectively. Both compounds were evidenced by the SIMPER test (Table 2 and Table 3) as the main contributors to the total dissimilarity of the HSs between both +UVB/−UVB and +M/−M plants, representing a cumulative contribution of 49.69 and 46.59%, respectively. Nevertheless, the percentages of these compounds were significantly different exclusively for the +UVB/−UVB group of samples, as evidenced in Table 2. Moreover, 1-undecene was higher in the HSs of the UVB-exposed samples (48.0% in +M/+UVB and 56.6% in −M/+UVB) than in those of the unirradiated ones (20.1% in +M/−UVB and 25.0% in –M/–UVB). Conversely, decanal reached the highest percentages in the –UVB samples (27.4 and 19.2% in +M/−UVB and −M/−UVB, respectively). Likewise, undecanal (4.2 and 3.4% in +M/−UVB and −M/−UVB, respectively) contributed significantly to the differences between +UVB and −UVB samples.

Non-terpene derivatives were the main chemical class of compounds detected in all the sample HSs, as they ranged between 62.4 and 76.7%. The samples exposed to UVB radiations showed higher amounts of this chemical class (70.1% in +M/+UVB, and 76.7% in −M/+UVB) than the unirradiated samples (69.9% in +M/−UVB and 62.4% in −M/−UVB). However, the differences in non-terpene percentages among all the samples were not statistically significant. Compounds 1-undecene and decanal were the most representative components belonging to this class, accounting for up to 56.6 and 27.4%, respectively. Both compounds were evidenced by the SIMPER test (Table 2 and Table 3) as the main contributors to the total dissimilarity of the HSs between both +UVB/−UVB and +M/−M plants, representing a cumulative contribution.

The only chemicals showing statistical differences among all the samples were apocarotenoids, which were detected only in the HS of the sample +M/+UVB in significant amounts (8.3%) and were totally represented by (*E*)-geranyl acetone.

**Figure 5:**
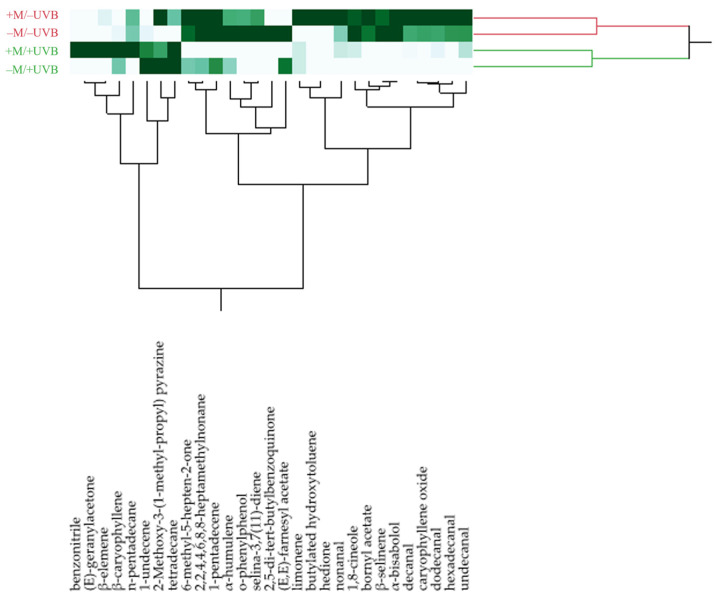
Heatmap of the HCA performed on the complete chemical composition of the headspaces (HSs) of the analysed lettuce samples exposed or not to UV-B radiation (+UVB, −UVB), and colonized or not by arbuscular mycorrhizal fungi (+M, −M). The heatmap is based on a green scale and a darker colour grade corresponds to a greater content of the respective compound.

The heatmap of the HCA performed on the complete chemical composition of the lettuce HSs is reported in Figure 5.

The four samples were grouped in two macro-clusters. The red cluster comprised the HSs of plants not exposed to UV-B treatment (+M/−UVB and −M/−UVB), whereas the green group was made up of the HSs of the two irradiated samples (+M/+UVB and −M/+UVB).

**Figure 6:**
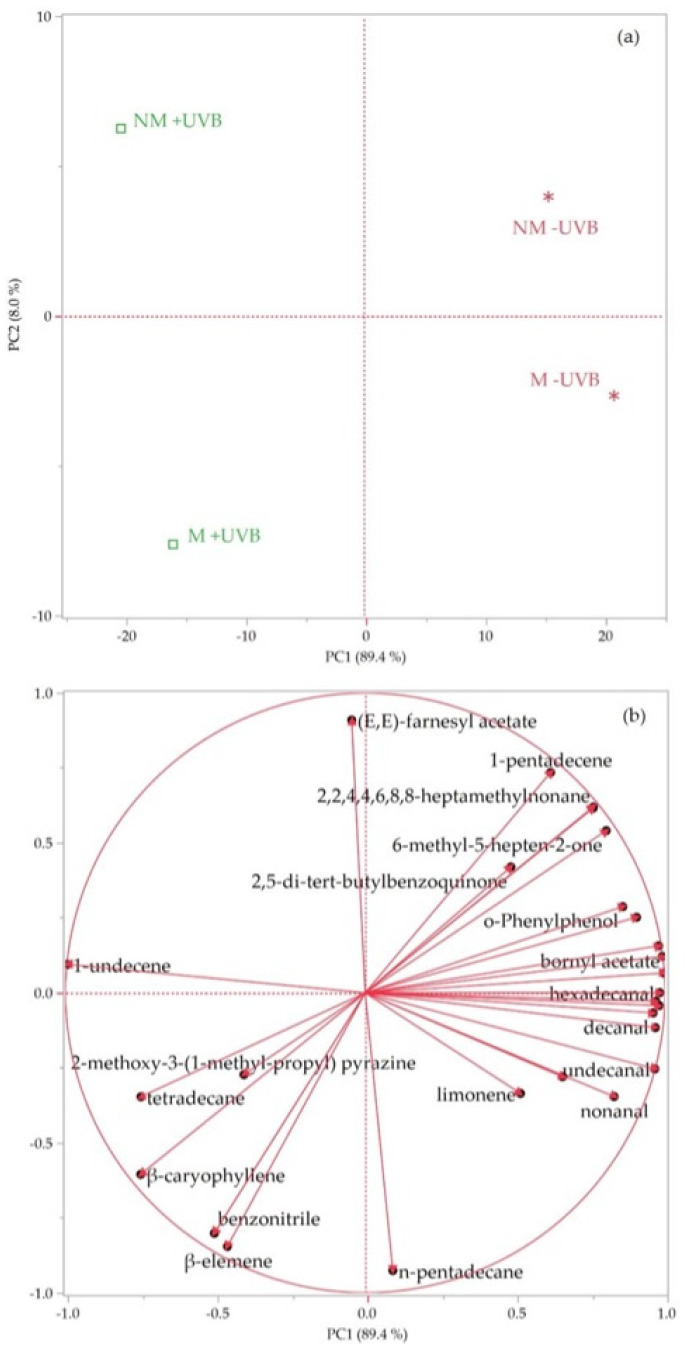
Score (**a**) and loading (**b**) plots of the PCA performed on the complete chemical composition of the HSs of the analysed *Lactuca sativa* plants exposed or not to UV-B radiation (+UVB, −UVB), and colonized or not by arbuscular mycorrhizal fungi (M, NM).

The score plot of the PCA (Figure 6a) evidenced the same partition of the HCA, as the samples of the red cluster were positioned in the right quadrants (PC1 > 0) and those belonging to the green group were in the left quadrants (PC1 < 0). As evidenced by the loading plot (Figure 6b), the positioning of the samples irradiated with UVB radiation was probably due to the greater percentages of 1-undecene, compared to the not irradiated samples. Moreover, within the green group, the sample −M/+UVB was plotted in the bottom quadrant (PC2 < 0) due to its higher percentage of 1-undecene than the sample +M/+UVB, which was plotted in the upper quadrant (PC2 > 0) given its greater content of β-caryophyllene. The positioning of the red samples, instead, was perhaps attributable to their major amounts of the aldehydes decanal and undecanal, vectors of which were clearly directed toward the right side of the loading plot. The same assumptions stood out from the heatmap of the two-way HCA, reported in Figure 5.

### 3.5. Feeding Preferences of Myzus persicae Aphids

**Figure 7:**
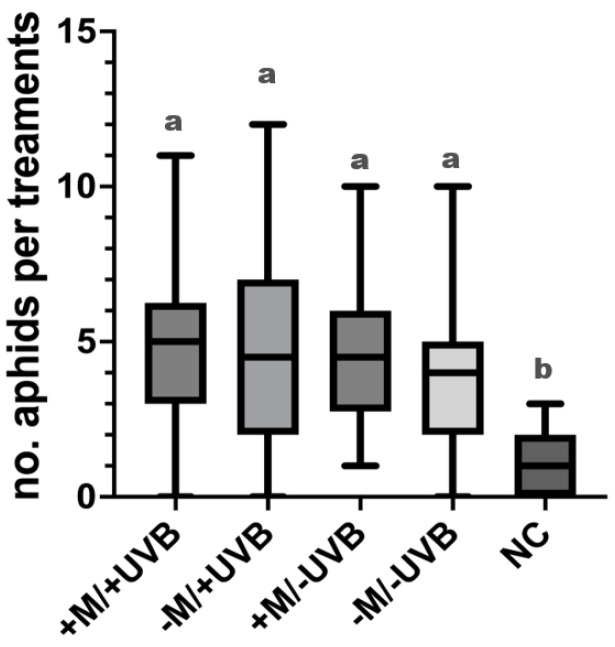
Boxplot showing the number of *Myzus persicae* aphids choosing *Lactuca sativa* plants exposed or not to UV-B radiation (+UVB, −UVB), and colonized or not by arbuscular mycorrhizal fungi (+M, −M). Each box plot indicates the median and its dispersion range (lower, upper quartile and extreme values, outliers). Above each boxplot, letters indicate significant differences among treatments (Kruskal–Wallis test, *p* > 0.05 followed by Steel–Dwass *p* < 0.05).

*Myzus persicae* choices were not random, except for the control (−M/−UVB), especially the no-choices (NC) and choices of aphids on lettuce plant exposed to UV-B and inoculated with AMF (+M/+UVB: *p* < 0.0001; −M/+UVB: *p* = 0.0327; +M/−UVB: *p* = 0.0252; −M/−UVB: *p* = 0.9669; NC: *p* < 0.0001). However, the only significant difference we detected was between aphids that made a choice, i.e., insects that chose a lettuce plant regardless of the treatment (+M/+UVB, −M/+UVB, +M/−UVB, −M/−UVB) and NC ones (*χ*^2^ = 48.4046; *d.f.* = 4; *p* < 0.0001) (Figure 7).

## 4. Discussion

We explored some aspects related to different conditions imposed on lettuce plants, in terms of UV-B irradiation and/or mycorrhization as well as the feeding preferences of aphids attacking *L. sativa*. UV-B irradiation can affect the biosynthesis of plant secondary metabolites, playing an important role in the acclimation of plants to various environmental conditions [52]. Among the different secondary metabolites, VOCs are interesting compounds involved in several functions, facilitating environmental stress adaptation and protection against oxidative stress due to their ability to neutralize ROS produced in stressful conditions [53]. Different studies reported changes in VOC metabolism under UV-B irradiation; however, the knowledge about these variations is very limited [54]. In our experiment, VOCs emitted by lettuce plants were affected by UV-B-exposure. In particular, the UV-B-exposed plants were characterized by lower percentages of sesquiterpene hydrocarbons if compared to unirradiated ones. Non-terpene derivatives showed an uneven behaviour, as the main detected component 1-undecene increased with UV-B-exposure, while the aldehydes decanal and undecanal were significantly higher in the unirradiated plants.

This is the first study investigating the variation of the volatilome composition in mycorrhizal plants of *L. sativa*. Mycorrhization did not lead to significant differences in the VOCs compositions of the studied samples. Such results are not consistent with the literature data, reporting drastic changes in secondary metabolism due to mycorrhizal symbiosis [15]. However, previous works have been carried out at later stages of mycorrhizal colonization in lettuce, 7–8 weeks post-inoculation, when the symbiosis is generally well established, with marked effects on plant secondary metabolism [55,56,57,58,59]. Here, lettuce plants were harvested three weeks post-inoculation, a limited period to detect strong variations in VOCs, although changes in the transcription patterns of genes related to secondary metabolism might already be induced by AMF, as shown in sunflower [60]. However, VOCs production may also depend on AMF and plant genotypes [61,62].

Mycorrhizal plants showed a significantly higher SD compared to non-mycorrhizal ones. Although UV-B radiation resulted in a decrease in SD, the simultaneous presence of AMF was able to restore the SD values as it had the control (–M/–UVB) ones. Indeed, it has been previously observed that UV-B radiation can induce photomorphogenic responses affecting stomatal development and functioning [63,64]. Therefore, the presence of AMF may improve plant performances by facilitating the re-establishment of SD control values. SD may be affected by various environmental stresses, including drought, whose detrimental effects are often buffered by AMF-triggered SD increases [65,66]. However, the mechanisms regulating stomatal development in mycorrhizal plants should be investigated, considering that these are controlled by plant brassinosteroids through the activation of protein kinases’ signalling system [67]. The increase of oxidative stress markers in –M/+UVB plants and their absence in +M/+UVB plants demonstrated a beneficial effect of AMF for the plant, which can limit ROS production, probably triggering positive antioxidant responses to deal with other environmental stressors [68]. In addition to the above, UV-B treatment caused callose deposition in leaves, which was basically undetectable in leaves of the other treatments. UV-B priming may enable the activation of a ‘cross-tolerance’ reaction for plants, activating stress responsive pathways such as those related to pathogen defence and responses to wounding [6,69]. Considering this, the induction of callose production could be interpreted as a helpful response for the plant, capable of prompting it to positively deal with stress. In support of this, mild increases in H_2_O_2_ through UV-B can act as a driver of adaptive responses to enhance the defence system and prevent deterioration by higher doses of stress [70,71].

Although UV-B exposure or AMF inoculation may affect multiple trophic levels, such as the interaction between treated plants and herbivore insects, aphid feeding choice did not change in our experiment [72,73,74,75]. These findings were not unexpected. Usually, sap-sucking insects, such as *M. persicae*, have a neutral or a positive response to AMF–plant interaction [76]. In our study, *M. persicae* did not show a preference among plants exposed or not to UV-B light and/or AMF. Therefore, we may assume a neutral response by the aphid to plants inoculated with AMF. Just as the UV-B treatment, AMF could alter the defensive chemicals of host plants [77], thus mediating a wide range of interactions between species, even though, in this study, the total volatile emission rate of the lettuce plants was not significantly influenced by mycorrhization. The neutral effect of UV-B treatment on aphid performance found in our study is consistent with those reported by Kuhlmann and Müller [73,74] and Kuśnierczyk et al. [78]. However, in their studies, the specialist cabbage aphid, *Brevicoryne brassicae* (L.) (Hemiptera: Aphididae), was found to be more sensitive to UV-B than the generalist species *M. persicae*. Kuhlmann and Müller [73] showed a sensitive reduction in the *B. brassicae* population on plants grown under high UV-B, whereas green peach aphid reproduction was not affected by UV-B exposure. Taken together, data in the published literature and our findings show that the two species have distinct sensitivities based on their specialization.

In addition, the major compounds of volatile profiles showing significant variations were not reported by the Pherobase website (https://www.pherobase.com/) as semiochemicals of *M. persicae*. Herbivore pest host plant selection behaviour can vary depending on qualitative and quantitative changes in plant VOCs. Overall, VOCs have the capacity to attract or repel herbivores, making it challenging to predict insect behaviour in reaction to plant-emitted chemicals [19,79,80,81]. A study conducted by Ahmed et al. [82] showed that *M. persicae* responded differently among seven cabbage cultivars. In particular, green peach aphids were highly attracted to cabbage cultivars rich in terpenes. The bulk of terpenes, when emitted from their related host plants, act as informative compounds for herbivore animals [83]. In our experiments the VOC profiles of lettuce plants were characterized by a low concentration of terpenes, which translates into a possible explanation of a non-feeding preference by aphids. On the other hand, an earlier study evidenced a reduced colonization by the specialist aphid *B. brassicae* in plants acclimatized to elevated levels of CO_2_ [84]. This response occurred in tandem with the emission of sufficient amounts of decanal and undecanal [84], both of which were found in the unirradiated plants of lettuce analysed in the present work. However, depending on the amount emitted and its relative abundance in the VOCs blend, it may display opposing behaviours on the same insect species [85,86].

Overall, the effects of UV-B-exposed and AMF-inoculated plants on *M. persicae* aphids may be highly dependent on the degree of specialization of the insect pest. The responses might also be affected by the plant developmental stage and the degree of insect adaptation to variations in host plant quality because of the treatments. 

## 5. Conclusions

This study explored the hypothesis that UV-B priming and AMF inoculation would change lettuce stomatal density, volatile emissions, and overall plant defence towards a polyphagous herbivorous pest. UV-B treatment increased the callose and oxidative stress markers while decreasing stomatal density, which was reversed in mycorrhizal lettuce plants. These findings reveal the beneficial effect of AMF, which triggered favourable antioxidant plant responses to UV-B exposure. VOCs composition was altered by the UV-B treatment, whereas it was not significantly affected by mycorrhization. However, variations in VOCs emissions, histochemical parameters, and SD did not have a significant impact on aphid infestation levels. Further investigations are needed to determine whether different UV-B irradiation doses and longer AMF inoculation times may alter the feeding preferences and overall trophic activity of *M*. *persicae*. It will be also essential to shed light on the potential ecological effects of altered VOC emissions, such as disruption of communication networks, in plants exposed to UV-B and inoculated with AMF.

## Figures and Tables

**Table 1:** Complete chemical composition of the headspaces emitted from *Lactuca sativa* samples exposed or not to UV-B radiation (+UVB, −UVB), and colonized or not by arbuscular mycorrhizal fungi (+M, −M).

Compound	l.r.i. ^1^	Class	Relative Abundance (%) ± SE (*n* = 4)			
			+M/−UVB	+M/+UVB	−M/−UVB	−M/+UVB
Benzonitrile	986	nt	^2^	1.4 ± 0.56	-	-
6-methyl-5-hepten-2-one	986	nt	1.3 ± 0.23	-	1.1 ± 0.22	0.7 ± 0.25
Limonene	1029	mh	2.6 ± 0.77	1.5 ± 0.5	1.4 ± 0.65	1.5 ± 0.44
1,8-cineole	1032	om	1.8 ± 0.96	0.7 ± 0.25	1.9 ± 1.07	0.3 ± 0.32
1-undecene	1091	nt	20.1 ± 5.55	48.0 ± 7.78	25.0 ± 4.36	56.6 ± 14.90
Nonanal	1105	nt	5.2 ± 0.71	2.6 ± 1.14	3.1 ± 0.88	2.0 ± 0.85
2-methoxy-3-(1-methyl-propyl) pyrazine	1175	pyr	1.4 ± 0.79	1.2 ± 1.17	0.8 ± 0.84	1.3 ± 1.34
Decanal	1206	nt	27.4 ± 7.33	8.5 ± 3.90	19.2 ± 8.00	8.0 ± 4.87
bornyl acetate	1286	om	0.7 ± 0.06	-	0.5 ± 0.31	-
Undecanal	1305	nt	4.2 ± 0.27	2.1 ± 0.65	3.4 ± 0.55	1.2 ± 1.18
2,2,4,4,6,8,8-heptamethylnonane	1322	nt	1.2 ± 0.26	-	1.2 ± 0.49	0.8 ± 0.52
β-elemene	1392	sh	1.5 ± 0.39	2.6 ± 0.78	1.5 ± 0.62	1.4 ± 0.23
*n*-tetradecane	1400	nt	1.1 ± 0.2	1.2 ± 0.13	1 ± 0.15	1.2 ± 0.17
Dodecanal	1409	nt	2.6 ± 0.18	1.6 ± 0.38	2.2 ± 0.43	1.5 ± 0.48
β-caryophyllene	1419	sh	8.6 ± 2.84	15.5 ± 8.97	9.4 ± 2.58	10.7 ± 5.34
I-geranylacetone	1453	ac	-	8.3 ± 3.24	-	-
α-humulene	1453	sh	11.1 ± 1.29	-	17.8 ± 5.75	7.1 ± 2.28
β-selinene	1486	sh	0.8 ± 0.14	-	0.9 ± 0.24	-
1-pentadecene	1493	nt	5.0 ± 1.85	3.5 ± 0.68	5.2 ± 1.35	4.7 ± 0.85
selina-3,7(11)-diene	1530	sh	0.8 ± 0.18	-	1.4 ± 0.44	-
caryophyllene oxide	1582	os	0.5 ± 0.18	-	0.4 ± 0.11	-
Hedione	1646	nt	0.6 ± 0.31	-	-	-
α-bisabolol	1685	os	0.5 ± 0.16	-	0.5 ± 0.1	-
hexadecanal	1816	nt	0.6 ± 0.14	-	0.4 ± 0.08	-
(*E,E*)-farnesyl acetate	1843	os	-	-	1.2 ± 0.43	1.0 ± 0.96
**Chemical Classes**	**l.r.i. ^1^**		**Relative Abundance (%) ± SE (*n* = 4)**			
			**+M/−UVB**	**+M/+UVB**	**−M/−UVB**	**−M/+UVB**
Monoterpene hydrocarbons (mh)			2.6 ± 0.77	1.5 ± 0.5	1.4 ± 0.65	1.5 ± 0.44
Oxygenated monoterpenes (om)			2.5 ± 0.96	0.7 ± 0.25	2.4 ± 0.94	0.3 ± 0.32
Sesquiterpene hydrocarbons (sh)			22.7 ± 3.75	18.2 ± 9.62	30.9 ± 6.49	19.2 ± 5.9
Oxygenated sesquiterpenes (os)			1.0 ± 0.34	-	2.1 ± 0.52	1.0 ± 0.96
Other non-terpene derivatives (nt)			69.9 ± 4.17	70.1 ± 7.48	62.4 ± 7.5	76.7 ± 7.18
Pyrazines (pyr)			1.4 ± 0.79	1.2 ± 1.17	0.8 ± 0.84	1.3 ± 1.34
Apocarotenoids (ac)			- B	8.3 ± 3.24 A	- B	- B
Total identified (%)			100.0 ± 0.01	100.0 ± 0.00	100.0 ± 0.01	100.0 ± 0.00

^1^ Linear retention indices determined on an HP-5MS capillary column. ^2^ Not detected. Superscript uppercases (A, B) indicates statistical differences among samples (Tukey’s HSD test, *p* < 0.05).

**Table 2:** Similarity percentages (SIMPER) test for the compositions of the headspaces emitted from *Lactuca sativa* samples exposed or not to UV-B radiation (+UVB and −UVB, respectively).

Compound	Average Dissimilarity	Individual Contribution (%)	Cumulative Contribution (%)
**1-undecene**	**15.88**	**32.11**	**32.11**
**Decanal**	**8.695**	**17.58**	**49.69**
**α-humulene**	**5.582**	**11.29**	**60.98**
β-caryophyllene	5.384	10.89	71.86
(***E***)-geranyl acetone	2.077	4.201	76.06
1-pentadecene	1.388	2.806	78.87
nonanal	1.282	2.593	81.46
**undecanal**	**1.263**	**2.555**	**84.02**
2-methoxy-3-(1-methyl-propyl) pyrazine	0.9025	1.825	85.84
1,8-cineole	0.7889	1.595	87.44
limonene	0.6703	1.355	88.79
β-elemene	0.5969	1.207	90
2,2,4,4,6,8,8-heptamethylnonane	0.5533	1.119	91.12
**dodecanal**	**0.5377**	**1.087**	**92.2**
**selina-3,7(11)-diene**	**0.5375**	**1.087**	**93.29**

Compounds that showed statistically differences among +UVB and −UVB are evidenced in bold.

**Table 3:** Similarity percentages (SIMPER) test for the compositions of the headspaces emitted from *Lactuca sativa* samples colonized or not by arbuscular mycorrhizal fungi (+M and −M, respectively)

Compound	Average Dissimilarity	Individual Contribution (%)	Cumulative Contribution (%)
1-undecene	12.37	29.18	29.18
decanal	7.374	17.4	46.59
β-caryophyllene	5.208	12.29	58.88
α-humulene	4.694	11.08	69.96
(***E***)-geranyl acetone	2.077	4.903	74.86
1-pentadecene	1.342	3.167	78.03
nonanal	1.187	2.802	80.83
undecanal	1.047	2.47	83.3
2-methoxy-3-(1-methyl-propyl) pyrazine	0.8819	2.081	85.38
1,8-cineole	0.7098	1.675	87.06
limonene	0.6746	1.592	88.65
β-elemene	0.615	1.451	90.1
(***E,E***)-farnesyl acetate	0.5462	1.289	91.39
dodecanal	0.4752	1.121	92.51
2,2,4,4,6,8,8-heptamethylnonane	0.4731	1.117	93.63

## Data Availability

Data are available from the corresponding author at reasonable request.
